# Morphological patterns of fetal lateral ventricular border irregularities: descriptive study

**DOI:** 10.1002/uog.70217

**Published:** 2026-04-15

**Authors:** E. Hadi, A. Sorotzkin, L. Haddad, E. Kassif, C. Hoffmann, S. Shrot, S. Shoob, A. Shariv, Y. Mizrachi, M. Levy, Z. Leibovitz, T. Lerman‐Sagie, L. Gindes

**Affiliations:** ^1^ Diagnostic Ultrasound Unit, The Institute of Obstetrical and Gynecological Imaging, Department of Obstetrics and Gynecology Sheba Medical Center Ramat Gan Israel; ^2^ Multidisciplinary Fetal Neurology Center, and Fetal Brain Research Center, Wolfson Medical Center Holon Israel; ^3^ The Gray Faculty of Medical and Health Sciences Tel Aviv University Tel Aviv Israel; ^4^ Ultrasound Unit, Department of Obstetrics and Gynecology Wolfson Medical Center Holon Israel; ^5^ Neuroradiology Unit, Department of Diagnostic Radiology Sheba Medical Center Ramat Gan Israel; ^6^ Fertility Unit, Wolfson Medical Center Holon Israel; ^7^ Prenatal Genetic Diagnosis Unit, Institute of Genetics, Tel Aviv Sourasky Medical Center Tel Aviv Israel; ^8^ Obstetrics and Gynecology Ultrasound Unit, Bnai‐Zion Medical Center Haifa Israel; ^9^ Rappaport Faculty of Medicine, Technion‐Israel Institute of Technology Haifa Israel

**Keywords:** brain disruption, fetal brain, lateral ventricles, malformations of cortical development, MRI, neurosonography

## Abstract

**Objectives:**

Alterations in the lateral ventricular borders have been documented in the prenatal diagnosis of certain fetal brain conditions. This study aimed to describe and classify the morphological patterns of lateral ventricular border irregularities (LVBI) and to discuss possible etiologies.

**Methods:**

This multicenter retrospective study reviewed all cases of prenatally diagnosed LVBI at three centers between January 2014 and December 2022. Neurosonography and fetal magnetic resonance imaging were used to determine the type of LVBI, its location and the presence of other ependymal abnormalities. Data regarding other prenatally diagnosed malformations, maternal TORCH serology, genetic testing, autopsy findings and postnatal outcomes were collected.

**Results:**

Sixty‐six fetuses were included in the analysis. Genetic testing was performed in 30/66 (45.5%) cases. Termination of pregnancy was elected in 33/66 (50.0%) of cases, and 28/66 (42.4%) were liveborn (mean ± SD age at postnatal neurodevelopmental follow‐up, 3.4 ± 1.2 years). Four main LVBI patterns were identified: protrusions (nodular or non‐nodular), indentations (round or wedge‐shaped), undulations and mixed. Nodular protrusions (19/66 (28.8%)) were observed with neuronal migration disorders (periventricular nodular heterotopia or tuberous sclerosis complex), whereas non‐nodular protrusions (4/66 (6.1%)) were observed in cases of disruptive injury (intraventricular hemorrhage or intrauterine fetal cytomegalovirus infection). Round indentations (7/66 (10.6%)) were observed in the context of porencephalic cysts, whereas wedge indentations (15/66 (22.7%)) were typically consistent with either periventricular venous hemorrhagic infarction or cleft (schizencephaly) with an abnormal ependymal lining. Undulating or mixed patterns (21/66 (31.8%)) were often observed in association with other malformations of cortical development.

**Conclusions:**

Characterizing the pattern of LVBI can provide a framework for describing fetal brain anomalies and suggesting their etiologies. These morphological configurations may represent different developmental or disruptive etiologies, but causal relationships require further study. © 2026 The Author(s). *Ultrasound in Obstetrics & Gynecology* published by John Wiley & Sons Ltd on behalf of International Society of Ultrasound in Obstetrics and Gynecology.

## INTRODUCTION

Ventriculomegaly is the most common sign of a fetal brain anomaly, although mild and isolated ventriculomegaly are often benign^1^, ^2^, ^3^, ^4^. A meticulous inspection of the ventricular lumen, ventricular wall and periventricular zone is mandatory in cases of fetal ventriculomegaly and is a valuable tool for etiological diagnosis^5^. Abnormal shape of the lateral ventricles, abnormal echogenicity of the periventricular zone and lateral ventricular border irregularities (LVBI) are suggestive of fetal brain pathology.

LVBI may originate from masses protruding into the ventricular lumen, such as subependymal nodules^6^, ^7^, ^8^, ^9^ or intraventricular blood clots adhering to the ventricular wall. Alternatively, they may stem from periventricular parenchymal destruction, as observed in cases of porencephalic cyst or schizencephaly^10^, ^11^. The underlying etiology can be classified as developmental (neurogenetic or syndromic), disruptive or idiopathic.

LVBI have been documented in various fetal brain conditions, including periventricular venous hemorrhagic infarction (PVHI), congenital cytomegalovirus (CMV) infection, periventricular nodular heterotopia (PNH) and tuberous sclerosis complex (TSC)^6^, ^7^, ^8^, ^9^, ^10^, ^11^, ^12^, ^13^. LVBI have been recognized as a significant intracranial imaging feature indicative of fetal malformations of cortical development (MCD), as highlighted in the studies of Malinger *et al*.^12^ and Lerman‐Sagie *et al*.^13^. While specific patterns of ventricular shape are known to be associated with specific fetal brain pathologies^6^, ^14^, ^15^, ^16^, such correlation has not been studied systematically in relation to LVBI.

The purpose of this study was to describe different patterns of LVBI within a multicenter cohort of prenatally detected cases and to discuss possible etiologies.

## METHODS

### Case identification and eligibility

We searched the electronic ultrasound and magnetic resonance imaging (MRI) databases of Wolfson Medical Center, Holon, Israel; Sheba Medical Center, Ramat Gan, Israel; and Bnai‐Zion Medical Center, Haifa, Israel, for examinations performed between 1 January 2014 and 31 December 2022 owing to suspected fetal anomalies or intrauterine infection. Searches were performed using the terms ‘ventricular border irregularity’, ‘ventricular wall irregularity’, ‘subependymal nodule’, ‘porencephaly’, ‘cyst’ and related terms.

Inclusion criteria were the prenatal detection of LVBI on targeted neurosonography, imaging that satisfied the International Society of Ultrasound in Obstetrics and Gynecology (ISUOG) guidelines for targeted neurosonography^17^ and available clinical data. Exclusion criteria were inadequate image quality, indeterminate lesions after expert panel review and missing clinical data.

### Imaging acquisition

Neurosonography was performed using GE Voluson systems (E6, E8 and E10; GE Healthcare, Zipf, Austria) with 2–6‐MHz transabdominal and/or 5–9‐ or 6–12‐MHz transvaginal probes, using a combined transabdominal and transvaginal approach for fetuses in vertex presentation. Fetal MRI studies were performed using 1.5‐Tesla or 3.0‐Tesla scanners with a phased‐array abdominal coil. Maternal sedation (5 mg diazepam) was administered sublingually prior to the MRI study. The protocol conformed to ISUOG recommendations and included acquisition of single‐shot fast spin‐echo T2‐weighted images in the axial, coronal and sagittal planes (3 mm slice thickness).

### Imaging analysis

**Figure 1:**
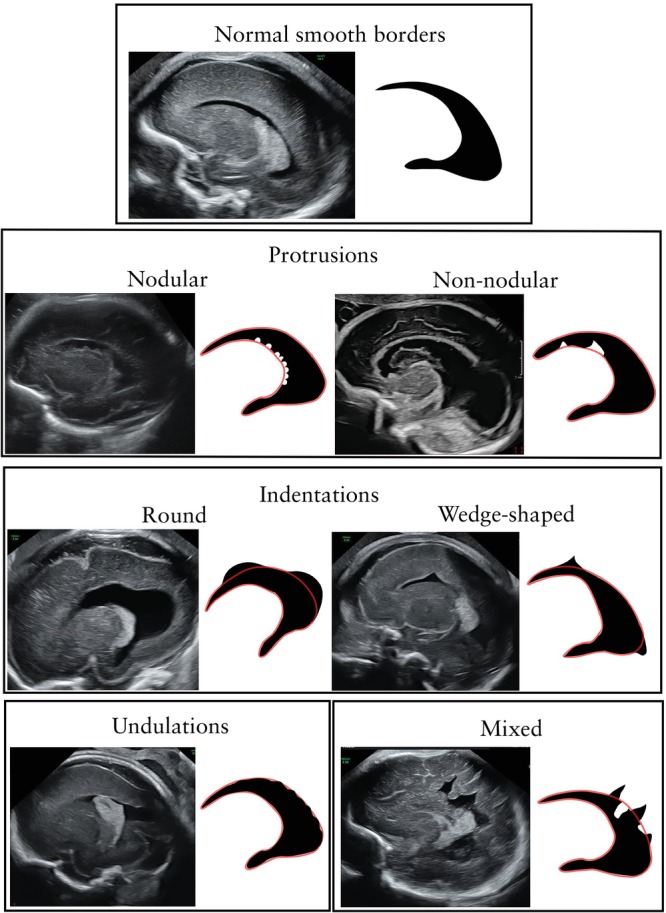
Schematic representations and corresponding ultrasound images of four main patterns of fetal lateral ventricular border irregularities (protrusion, indentation, undulation and mixed) compared with normal smooth lateral ventricular borders.

Ultrasound scans and magnetic resonance images of all included cases were reviewed by at least three experts from our team (E.H., L.H., T.L.‐S., Z.L. and L.G.), who discussed each case and agreed on the specific LVBI pattern. Each LVBI case was assigned to one of four morphological patterns based on the contour of the lateral ventricular wall (Figure 1): protruding pattern (a focal bulge extending from the ventricular border into its lumen, further classified as nodular or non‐nodular); indented pattern (a focal concavity below the expected border, further classified as round or wedge‐shaped); undulating pattern (a wave‐like, alternating pattern that did not meet the criteria for either protrusion or indentation); or mixed pattern (a combination of two or more of the above patterns). The co‐occurrence of undulation with nodular protrusions was not classified as mixed pattern, as this appearance was instead considered within the PNH spectrum.

LVBI were evaluated for additional characteristics, if applicable, which included: echogenicity of protrusions (isoechogenic, hypoechogenic or hyperechogenic); number of protrusions (single (≤ 4) or multiple (> 4)); contiguity (contiguous or noncontiguous in the case of multiple protrusions); location (anterior/frontal (anterior to the foramen of Monro), parietal (posterior to the foramen of Monro and superior to the upper border of the thalamus), posterior/occipital (inferior to the upper border of the thalamus), temporal or diffuse); and ependymal changes (hyperechogenic, thick or serrated (irregular)).

Each LVBI case was assessed for additional central nervous system (CNS) findings, including cortical malformations, corpus callosum disorders, absent septum pellucidum, posterior fossa anomalies and abnormal head circumference. Extra‐CNS malformations were also noted. Ventriculomegaly was defined according to the ISUOG guidelines as ventricular width ≥ 10 mm at the level of the atrium^18^. MCD were defined according to the updated classification^11^. Prenatal investigation results, including maternal TORCH serology and genetic testing, were collected.

A case was classified as isolated if no additional CNS or extra‐CNS anomalies were identified. A case was classified as non‐isolated if any additional CNS or extra‐CNS anomaly was present. For classification purposes, only structural abnormalities considered clinically significant were included as associated findings. Benign findings (e.g. choroid plexus cysts) and findings secondary to IVH (e.g. mild asymmetric ventriculomegaly, deviated cavum septi pellucidi, changes in ventricular configuration) were not considered to be additional abnormalities, as they were presumed to result from, or be closely linked to, the underlying LVBI pattern.

Postnatal work‐up for live births and autopsy results, when available, were documented. Medical reports from the pediatric neurology clinic follow‐up assessment, including comprehensive neurodevelopmental assessment by a pediatric neurologist (T.L.‐S.) and postnatal imaging, were reviewed. Therapeutic interventions, anticonvulsant treatment, educational interventions and special assistance requirements were also recorded.

A parental telephone interview was conducted using the Ages and Stages Questionnaire, 3^rd^ Edition (ASQ‐3)^19^, translated into Hebrew. Normal development was defined as achievement of a score above the age‐adjusted cut‐off value for all five assessed domains. For cases with scores near or below the cut‐off, study conclusions were supplemented by medical documentation and neurodevelopmental follow‐up records rather than relying solely on ASQ‐3 scores.

Etiologies of CNS anomalies were defined and categorized as follows: a neurogenetic etiology was assigned to cases with genetic variants involving mainly the CNS; a syndromic etiology was assigned to cases with genetic variants presenting with both CNS and extra‐CNS anomalies; a disruptive etiology was assigned to cases with CNS anomalies attributed to infections or ischemic/hemorrhagic events; and an idiopathic etiology was assigned to cases with isolated LVBI (e.g. single PNH) lacking genetic, infectious and ischemic/hemorrhagic findings despite a thorough investigation.

Statistical analysis was performed using SPSS version 28 (IBM Corp., Armonk, NY, USA). Continuous variables were presented as mean ± SD or mean (95% CI) and compared using the Student's *t*‐test, and categorical variables were presented as *n* (%) and compared using the chi‐square or Fisher's exact test, as appropriate.

The study was approved by the ethical committee of each of the three institutions (0061‐22‐WOMC, 9700‐22‐SMC, 0158‐22‐BNZ).

## RESULTS

### Study cohort and baseline characteristics

Of the 77 fetuses identified with LVBI on prenatal ultrasound, 10 were excluded because of inadequate imaging quality and one was excluded because the findings did not conform to any LVBI pattern. Thus, the final cohort included 66 fetuses. Clinical and imaging characteristics of the study population are summarized in Table 1.

**Table 1:** Clinical, imaging, genetic and outcome characteristics of the study population of 66 fetuses with lateral ventricular border irregularities

Characteristic	Value
Maternal age (years)	31.8 (30.4–33.2)
Gestational age at initial neurosonogram (weeks)	30.1 (28.9–31.3)
Prenatal MRI studies	42 (63.6)
Gestational age at MRI (weeks)	31.2 (30.0–32.4)
Genetic testing	
CMA	26 (39.4)
WES	13 (19.7)
Karyotyping	4 (6.1)
Not tested	36 (54.5)
Pregnancy outcome	
Live birth	28 (42.4)
Termination of pregnancy	33 (50.0)
Lost to follow‐up	3 (4.5)
Intrauterine fetal demise	2 (3.0)

Data are given as mean (95% CI) or *n* (%). CMA, chromosomal microarray analysis; MRI, magnetic resonance imaging; WES, whole‐exome sequencing.

LVBI was the primary indication for referral in nine (13.6%) cases. Other referral indications, which were not mutually exclusive, included ventricular abnormalities other than LVBI (ventriculomegaly, ventricular asymmetry or a dysmorphic ventricle) in 31 (47.0%) cases, suspected CNS anomalies in 18 (27.3%) cases, suspected extra‐CNS anomalies in 11 (16.7%) cases and miscellaneous indications in five (7.6%) cases. Twenty‐eight (42.4%) cases were liveborn, 33 (50.0%) underwent termination of pregnancy (TOP), two (3.0%) ended in perinatal death and three (4.5%) were lost to follow‐up. To assess neurodevelopmental outcomes, liveborn infants were examined by a pediatric neurologist (*n* = 7) or evaluated using the ASQ‐3 via parental telephone interview (*n* = 21). The mean ± SD age at any neurodevelopmental assessment (neurological examination or ASQ‐3) was 3.4 ± 1.2 years. Obstetric characteristics and neurodevelopmental outcomes of the liveborn infants are presented in Table 2.

**Table 2:** Obstetric characteristics and neurodevelopmental outcomes of 28 liveborn cases with lateral ventricular border irregularities

Characteristic	Value
Gestational age at delivery (weeks)	37.5 (36.4–38.6)
Birth weight (g)	2862.0 (2599.0–3124.0)
Neurodevelopmental outcome*	
Normal development	22 (78.6)
Neurodevelopmental delay	6 (21.4)
Motor impairment	4 (14.3)
Epilepsy	5 (17.9)

Data are given as mean (95% CI) or *n* (%).

* Mean ± SD age at neurodevelopmental assessment was 3.4 ± 1.2 years.

### Ultrasound image analysis and pattern distribution

In 46/66 (69.7%) cases, LVBI were observed with ventriculomegaly. In the other 20 (30.3%) cases, the ventricles were prominent (< 10 mm and ≥ 8 mm) or dysmorphic. In cases of bilateral ventriculomegaly, LVBI were typically observed on both sides. When the ventricles were asymmetric, LVBI were ipsilateral to the larger side.

**Figure 2:**
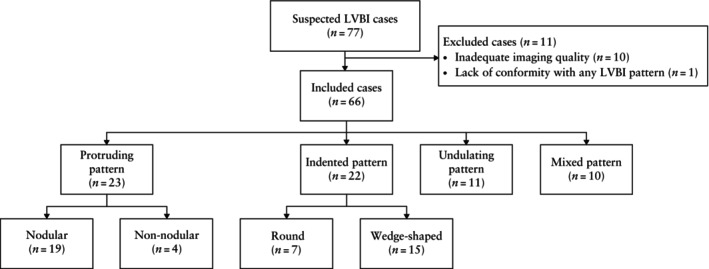
Flowchart showing inclusion of cases with fetal lateral ventricular border irregularities (LVBI), and pattern distribution.

LVBI were classified as protrusions in 23 (34.8%) fetuses, indentations in 22 (33.3%), undulations in 11 (16.7%) and mixed in 10 (15.2%) (Figure 2). The imaging characteristics of each LVBI pattern and their associations with neuronal migration disorders are described below.

### Protruding LVBI pattern

There were 23 (34.8%) cases with protruding LVBI, of which 19 were classified as nodular and four as non‐nodular. Among the nodular protrusion cases (NP1–19), several subtypes were identified (Table S1).

**Figure 3:**
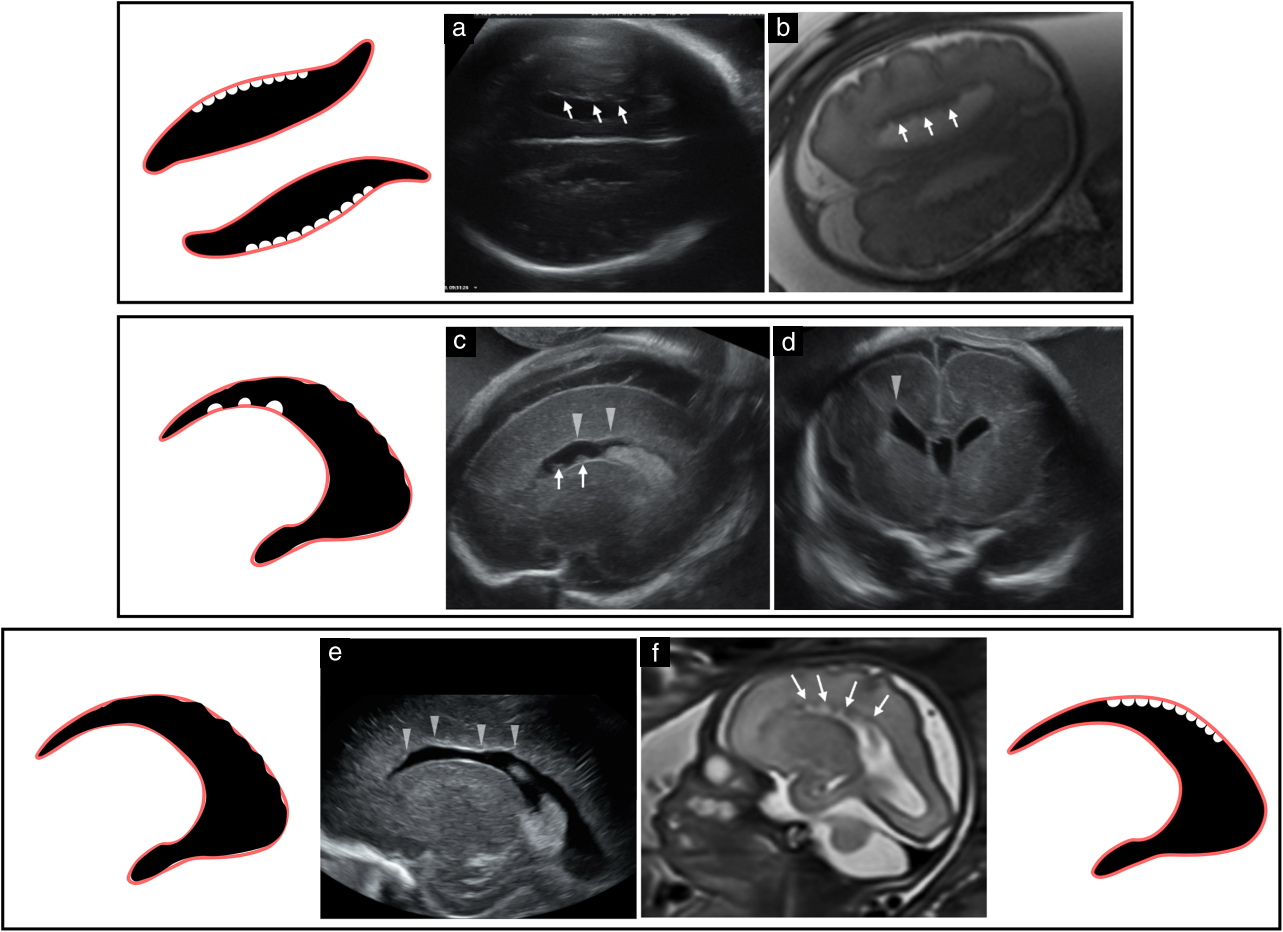
Nodular protrusion patterns of lateral ventricular border irregularities representing periventricular nodular heterotopia in three female fetuses with *FLNA* gene mutation, and corresponding schematic diagrams. (a,b) Case NP1 at 37 weeks: (a) axial transvaginal ultrasound shows multiple diffuse, bilateral, contiguous small nodular protrusions along the lateral ventricular border (LVB) (arrows) with intermediate echogenicity; and (b) corresponding T2‐weighted magnetic resonance imaging (MRI) shows low‐signal nodules isointense to cortex (arrows). (c,d) Case NP3 at 25 weeks: (c) sagittal transvaginal ultrasound shows small nodular protrusions along lower LVB (arrows) and undulating pattern along upper LVB (arrowheads); and (d) coronal transvaginal ultrasound demonstrates a squared anterior horn (arrowhead). (e,f) Case NP4: (e) sagittal transvaginal ultrasound at 26 weeks shows undulating LVB pattern (arrowheads); and (f) T2‐weighted MRI at 28 weeks demonstrates multiple small nodular protrusions along the upper LVB (arrows).

Four cases (NP1–4) presented with multiple diffuse contiguous small nodules along the ventricular border, showing intermediate echogenicity on ultrasound and T2 hypointensity similar to that of the cortex on MRI, consistent with PNH. These nodules were seen only in female fetuses and were accompanied by prominent, square‐shaped anterior horns and posterior fossa enlargement, including cases related to arachnoid cyst (Case NP3) and mega cisterna magna. No other CNS anomalies were detected. In two of these fetuses (NP3 and NP4), an additional undulating LVBI pattern was noted on ultrasound (Figure 3), and *FLNA* gene mutations were confirmed. In another case (NP2), a maternal *FLNA* mutation was identified, while the remaining case (NP1) was evaluated based on imaging findings alone. All four liveborn infants later presented with normal neurodevelopment.

**Figure 4:**
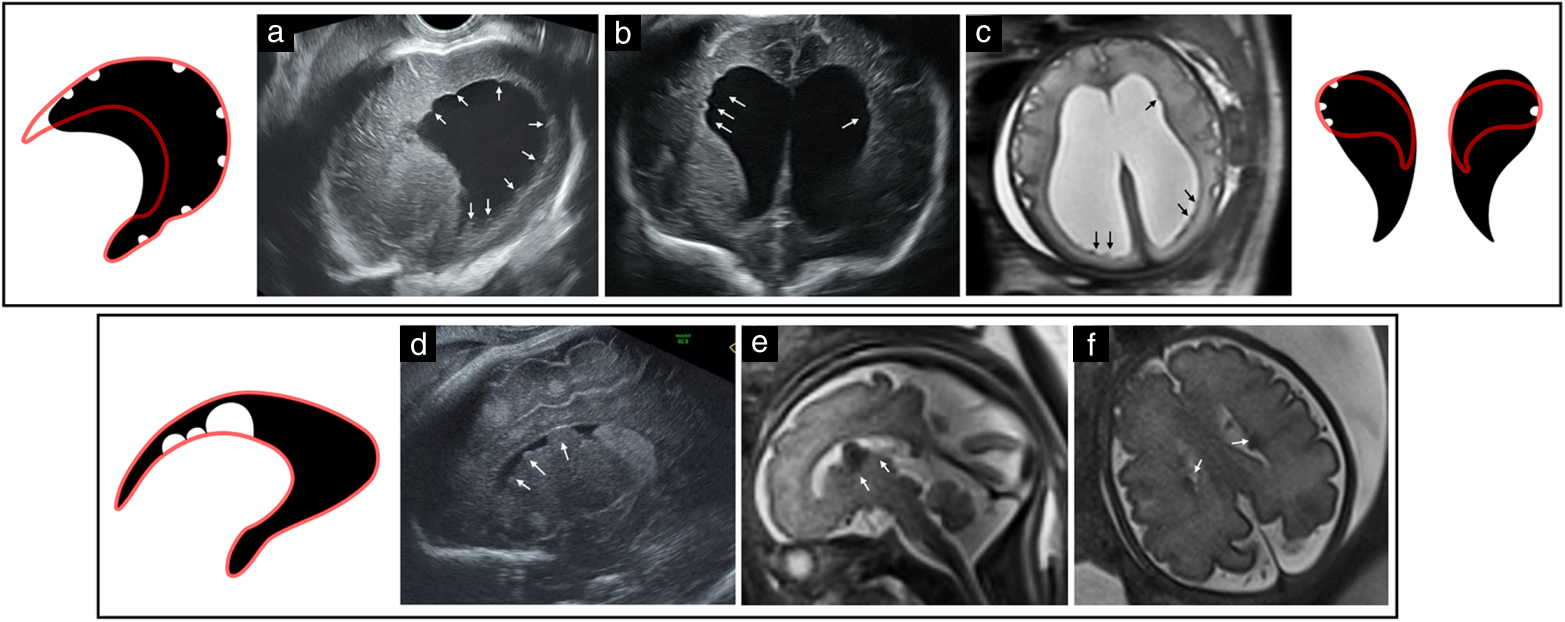
Nodular protrusion patterns of lateral ventricular border irregularities and corresponding schematic diagrams. (a–c) Case NP5, diagnosed prenatally with periventricular nodular heterotopia and hemorrhage‐related aqueductal stenosis leading to hydrocephalus. Sagittal (a) and coronal (b) transvaginal ultrasound at 33 weeks shows multiple small diffuse nodular protrusions (arrows). (c) Axial T2‐weighted magnetic resonance imaging (MRI) confirms nodular protrusions (arrows). Susceptibility artifact on heme‐sensitive MRI sequence (not shown) suggested early intraventricular hemorrhage as probable etiology for aqueductal stenosis. (d–f) Case NP12, diagnosed prenatally with tuberous sclerosis complex. (d) Sagittal transvaginal ultrasound at 32 weeks shows nodular protrusions with a large nodule at the caudothalamic groove (arrows). Cortical and subcortical echogenic foci consistent with tubers are also seen. Sagittal (e) and axial (f) T2‐weighted MRI shows hypointense subependymal nodules (arrows).

One case (NP5) presented with multiple diffuse noncontiguous small nodules with intermediate echogenicity, consistent with PNH. This fetus had aqueductal stenosis and severe ventriculomegaly. Hemosiderin deposits on susceptibility‐weighted MRI suggested intraventricular hemorrhage (IVH) as the underlying etiology for aqueductal stenosis (Figure 4a–c). At 27 months, the infant showed normal neurodevelopment and a normal electroencephalogram.

Six cases (NP6–11) presented with multiple asymmetric small nodules with intermediate echogenicity, consistent with PNH. The nodules had an asymmetric distribution, with side preference (laterality) and focal localization along the ventricular borders, typically restricted to anterior, parietal or posterior regions. Imaging findings in all cases suggested a neurogenetic or syndromic etiology, with MCD present in four cases, extra‐CNS abnormalities in three cases and extremely elevated alpha‐fetoprotein levels in one case. In two cases (NP7 and NP8), abnormal sulcation, characterized by atypical sulcal depth and orientation without clear cortical thickening or irregularity, was observed above the nodules. In one of these cases (NP8), dysgyria was confirmed on autopsy after TOP. In another case (NP11) that underwent TOP, subcortical heterotopia was observed on autopsy. A diagnosis of Mowat–Wilson syndrome was confirmed in a separate case (NP10).

Three cases (NP17–19) presented with single nodules consistent with PNH. Isolated nodules were observed in two cases, of which one infant showed normal neurodevelopment and the other was lost to follow‐up. In a third case, a single non‐isolated nodule associated with clubfoot was observed. This pregnancy ended in intrauterine fetal demise at 35 weeks, with no autopsy.

Five cases (NP12–16) presented with multiple nodules of varying size, isoechogenic to the adjacent parenchyma on ultrasound and T2‐hypointense on MRI (Figure 4d–f). These represented non‐isolated TSC‐related subependymal nodules (SEN), all accompanied by cortical tubers and cardiac rhabdomyomas. The larger nodules (up to 15 mm in diameter) tended to be contiguous and appeared as ridge‐like protrusions, often at the level of the foramen of Monro.

**Figure 5:**
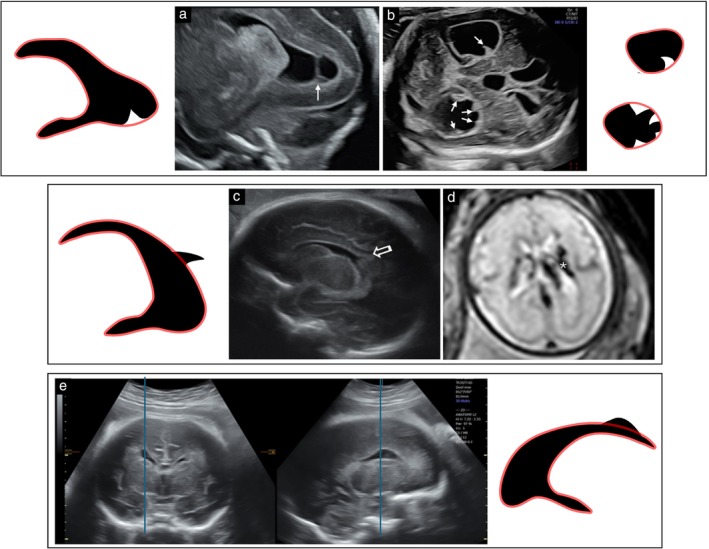
Patterns of lateral ventricular border irregularities (LVBI) of disruptive etiology and corresponding schematic diagrams. (a) Case NNP4, diagnosed prenatally with cytomegalovirus (CMV). Sagittal transvaginal ultrasound at 23 weeks shows a hyperechogenic, non‐nodular protrusion at base of an occipital horn adhesion (arrow) with periventricular hyperechogenic halo. (b) Case NNP1, diagnosed prenatally with acute/subacute intraventricular hemorrhage. Axial transvaginal ultrasound at 35 weeks shows multiple non‐nodular hyperechogenic protrusions of varying size and shape (arrows), consistent with blood clots adherent to ventricular walls. (c,d) Case WI8, diagnosed prenatally with remote periventricular venous hemorrhagic infarction (PVHI). (c) Sagittal transvaginal ultrasound at 35 weeks shows wedge‐shaped indentation (arrow), with thickened, hyperechogenic border. (d) Axial heme‐sensitive magnetic resonance imaging at 35 weeks confirms periventricular blood remnants (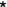). (e) Case WI2, diagnosed prenatally with remote PVHI. Three‐dimensional ultrasound at 33 weeks shows wedge‐shaped indentation with anterior horn distortion, consistent with porencephaly.

Four cases (NNP1–4) presented with non‐nodular protrusions of varying size and shape, which appeared hyperechogenic (Table S2, Figure 5a,b). These protrusions corresponded to adherent blood clots or inflammatory adhesions (due to IVH or CMV infection, respectively) and were accompanied by ependymal abnormalities (ventricular lining that was hyperechogenic, thickened or serrated).

### Indented LVBI pattern

**Figure 6:**
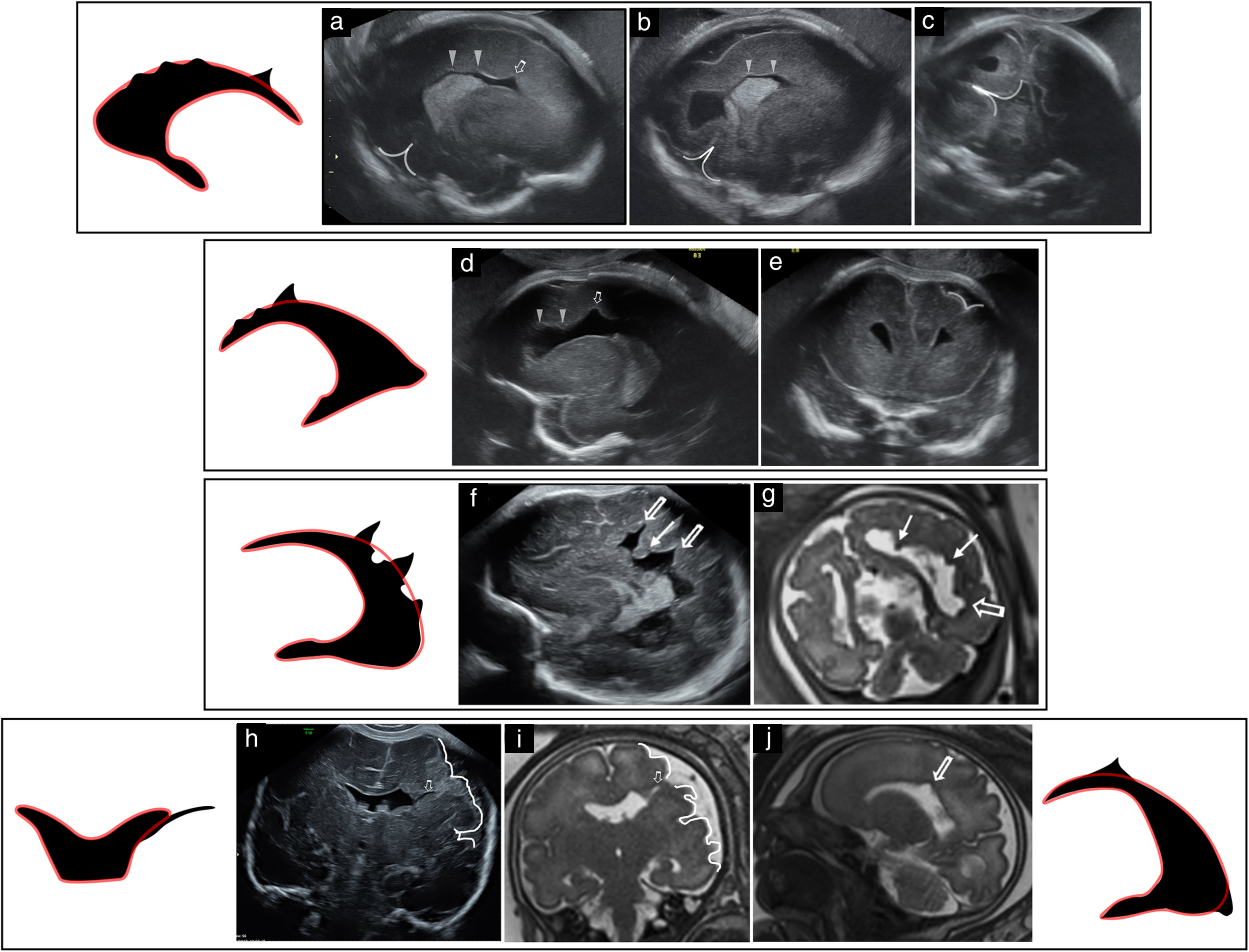
Patterns of lateral ventricular border irregularities (LVBI) observed with malformations of cortical development, and corresponding schematic diagrams. (a–c) Case M2, diagnosed prenatally with atretic posterior encephalocele. Sagittal (a,b) and coronal (c) transvaginal ultrasound at 24 weeks shows a mixed LVBI pattern including undulations (arrow heads), wedge‐shaped indentation (hollow arrow) and abnormal sulcation (curved line). Autopsy demonstrated periventricular nodular heterotopia (PNH) and subcortical heterotopia. (d,e) Case M1. (d) Sagittal transvaginal ultrasound at 24 weeks shows mixed LVBI pattern, including undulations (arrow heads) and wedge‐shaped indentation (hollow arrow). (e) Coronal transvaginal ultrasound at 24 weeks shows abnormal sulcation overlying ventricular border (curved line). Associated anomalies in this female fetus, including agenesis of the corpus callosum and retinal coloboma, suggest Aicardi syndrome. (f,g) Case M3. (f) Sagittal transvaginal ultrasound at 35 weeks shows diffuse mixed LVBI pattern with wedge‐shaped indentations (hollow arrows) and nodular protrusions (arrows). (g) Coronal T2‐weighted magnetic resonance imaging (MRI) at 35 weeks shows corresponding nodules isointense to cortex (arrows), consistent with PNH, and wedge‐shaped indentations (hollow arrow). Additional brain malformations and retinal coloboma in this female fetus support a diagnosis of Aicardi syndrome. (h–j) Case WI13. (h) Coronal transvaginal ultrasound at 32 weeks shows a deep wedge‐shaped indentation (hollow arrow), extending into unilateral cleft schizencephaly. (i,j) Coronal T2‐weighted MRI at 32 weeks (i) better delineates the transmantle cleft (hollow arrow), with a corresponding wedge‐shaped indentation in the sagittal view (j) (hollow arrow). Ipsilateral perisylvian polymicrogyria is evident (curved line).

Indented LVBI, indicating a localized loss of periventricular parenchyma, were noted in 22 (33.3%) cases. These indentations were further characterized as wedge‐shaped (15 cases; WI1–15) or round (seven cases; RI1–7). Wedge‐shaped indentations (Table S3) were observed with PVHI in 10 cases. The adjacent ependyma appeared hyperechogenic and occasionally serrated, consistent with a disruptive injury. Small wedge‐shaped indentations, sometimes seen alongside other signs of IVH, represented limited PVHI (five cases; WI1–5), while larger wedge‐shaped indentations were observed with more extensive parenchymal damage (e.g. Wallerian degeneration or porencephalic cysts) (five cases; WI6–10) (Figure 5c–e). Wedge‐shaped indentations were also observed with MCD (5 cases; WI11–15). Notably, in these cases, clefts (schizencephaly) or polymicrogyria (PMG) were often identified in the cortex just above the indentation (Figure 6h–j).

Round indentations (seven cases; RI1–7) (Table S4) were consistently indicative of porencephalic or periventricular cysts and occurred concomitantly with PVHI (four cases) or intrauterine CMV infection (three cases). Ependymal abnormalities, including hyperechogenicity, serrations and thickening, were observed. Additional imaging findings helped with the differential diagnosis, e.g. periventricular calcifications and abnormal lamination supported the diagnosis of CMV infection.

### Undulating LVBI pattern

An undulating pattern was observed in 11 (16.7%) cases (U1–11) (Table S5). Of these, six were diffuse undulations and five were focal. All six diffuse undulation cases (U1–6) were non‐isolated and were accompanied by MCD, including PMG‐like cortex in three fetuses and abnormal lamination in two fetuses. Portmortem examination confirmed PMG in one case and cobblestone malformation in another. Additional CNS anomalies (involving the cerebellum, brainstem or midline structures) were present in all these cases, and some also presented with extra‐CNS malformations. Four cases underwent TOP and two cases were liveborn, later presenting with severe global neurodevelopmental delay. Postnatal genetic testing in one of these cases (U3) revealed a pathogenic *FLVCR2* mutation, consistent with Fowler syndrome (proliferative vasculopathy and hydranencephaly). Of the five cases that showed focal undulations (U7–11), four had MCD and one was isolated.

### Mixed LVBI pattern

A mixed pattern was identified in 10 (15.2%) cases (Table S6). All cases showed additional CNS anomalies of either disruptive or developmental origin. In six cases (M1–6), the pattern was diffuse and accompanied by extra‐CNS anomalies, suggesting a syndromic or infectious etiology; all six of these cases underwent TOP (Figure 6a–g). In four cases (M7–10), the mixed LVBI pattern appeared focal in distribution; however, notable adjacent parenchymal or cortical abnormalities were also present. Three of these cases underwent TOP and in one case the fetus was liveborn and had quadriplegic cerebral palsy and borderline intelligence.

### LVBI pattern distribution

LVBI were isolated in 19.7% (13/66) of our cohort and non‐isolated in 80.3% (53/66). Table 3 summarizes the distribution of cases across LVBI patterns, conditions observed with LVBI, possible etiologies and the availability of genetic and outcome data.

**Table 3:** Summary of lateral ventricular border irregularity (LVBI) patterns, including numbers of cases, conditions observed with LVBI, possible etiologies, genetic testing and available outcome data

LVBI pattern	Cases (*n*)	Conditions observed with LVBI	Possible etiology	Genetic testing (*n/N*)	Available outcome data (*n/N*)
Nodular protrusions	19				
Single* small nodularprotrusions	3	PNH	Variable	0/3	2/3
Multiple diffuse small nodularprotrusions	5	PNH	*FLNA* mutation, early hydrocephalus	3/5	4/5
Multiple asymmetric smallnodular protrusions	6	PNH	May be associated with other MCD and genetic syndromes	3/6	4/6
Multiple nodular protrusions of varying size	5	Subependymal nodules	TSC	4/5	3/5
Non‐nodular protrusions (varying size and shape)	4	Blood clots / adhesions	Disruptive (IVH, CMV infection)	1/4	3/4
Round indentations	7	Parenchymal loss (porencephaly)	Disruptive (PVHI, CMV infection)	2/7	3/7
Wedge indentations	15	Parenchymal loss or MCD	PVHI, MCD	11/15	11/15
Undulations	11	Parenchymal loss or MCD	Disruptive or developmental	5/11	8/11
Mixed	10	Parenchymal loss or MCD	Disruptive or developmental	3/10	6/10

* Single defined as ≤ 4 nodular protrusions. CMV, cytomegalovirus; IVH, intraventricular hemorrhage; MCD, malformations of cortical development; PNH, periventricular nodular heterotopia; PVHI, periventricular venous hemorrhagic infarction; TSC, tuberous sclerosis complex.

**Figure 7:**
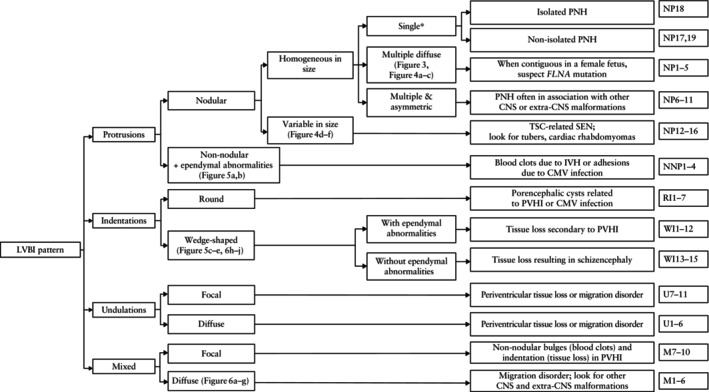
Investigation flowchart linking patterns of lateral ventricular border irregularities (LVBI) with characteristic imaging findings and outlining potential mechanisms and etiologies. This figure serves as an educational visual summary rather than a diagnostic algorithm. Case numbers are listed on the right. *Single defined as ≤ 4 nodular protrusions. CMV, cytomegalovirus; CNS, central nervous system; IVH, intraventricular hemorrhage; PNH, periventricular nodular heterotopia; PVHI, periventricular venous hemorrhagic infarction; SEN, subependymal nodules; TSC, tuberous sclerosis complex.

Figure 7 presents an investigation flowchart linking LVBI patterns with characteristic imaging findings and outlining potential mechanisms and etiologies.

### Gestational age and LVBI patterns

Gestational age at the first neurosonogram ranged from 18.4 to 38.6 weeks. Undulating and mixed LVBI patterns were identified earliest (median gestational age, 27.4 weeks), whereas wedge‐shaped indentations were detected later (median gestational age, 32.6 weeks). Overall, 38% of cases of LVBI were diagnosed in the second trimester (Table S7).

### Role of magnetic resonance imaging in refining pattern‐based diagnosis

Prenatal MRI was performed in 42 (63.6%) fetuses, typically at 31–32 weeks' gestation, within a short interval after the ultrasound examination. MRI findings showed 98% concordance with ultrasound findings in classifying the LVBI pattern and confirming associated malformations. MRI added diagnostic confidence in several ways: (1) by depicting T2‐hypointense nodular protrusions, supporting the diagnosis of PNH (MRI clarified an ‘undulating’ ultrasound impression as definitive PNH in two cases); (2) by revealing periventricular T2‐hypointensities along the indentations, confirming remote IVH‐PVHI when blood clots were no longer visible on ultrasound; and (3) by demonstrating periventricular white‐matter T2 signal changes supporting parenchymal involvement in CMV infection.

### Genetic analysis

Genetic investigations were performed in 30 cases (27 prenatally, two postnatally and one after TOP). Karyotype analysis (*n* = 4) and chromosomal microarray analysis (*n* = 26) were normal. Whole‐exome sequencing identified pathogenic variants in 7/13 (53.8%) cases, including two vasculopathies (*FLVCR2* (case U3) and *COL4A1* (case WI9)), and five variants associated with MCD (*TUBB3* (case U9), *FLNA* (cases NP3 and NP4), *MED13L* (case NP10) and *CREBBP* (case WI10)). In one additional case, a maternal *FLNA* mutation was found (case NP2). In cases with identified pathogenic genetic variants, LVBI were predominantly multifocal or diffuse and often observed with other CNS findings.

## DISCUSSION

This study describes four main morphologic patterns of LVBI. These four main types and their subtypes provide a framework for describing fetal CNS anomalies and suggesting their etiologies. The underlying etiology can be either developmental (e.g. PNH, TSC) or disruptive (e.g. PVHI, CMV). Importantly, nearly 40% of LVBI cases were detected during routine second‐trimester anatomy scans, highlighting the value of closely inspecting ventricular borders.

Small nodular protrusions were one of the most frequent LVBI subtypes in our series and were consistently observed with PNH. On prenatal ultrasound, these protrusions may appear as subtle undulations, whereas MRI confirms their nodular morphology. PNH occurs when neural stem cells lining the ventricles fail to migrate toward the cortex^20^, ^21^. This impaired neural migration can result from intrinsic cellular motility defects or it can be secondary to ependymal denudation; mutations in genes such as *FLNA* or *ARFGEF2* have demonstrated both mechanisms^22^, ^23^, ^24^. In addition, early obstructive hydrocephalus can mechanically denude the germinal epithelium, leading to heterotopia^25^. Multiple asymmetric nodules often coexist with other MCD and structural brain anomalies^26^, ^27^, ^28^, ^29^, ^30^, ^31^, ^32^. Recognition of this pattern should prompt a careful survey of the overlying cortex and subcortical region for additional anomalies. Multiple symmetric contiguous nodules are linked to *FLNA* mutations^6^, ^7^, ^8^ and an increased risk of epilepsy^32^, ^33^. Isolated nodular protrusions were less commonly diagnosed.

Multiple nodular protrusions of varying size that are isoechogenic to the surrounding parenchyma corresponded to TSC‐related SEN. These lesions were invariably located near the caudate nucleus or thalamus; notably, four of the five largest nodules were clustered in the caudothalamic groove, adjacent to the foramen of Monro. This signature location^9^, ^34^, ^35^, together with the presence of cortical or subcortical tubers and fetal cardiac rhabdomyomas, constitutes a reliable prenatal imaging triad for diagnosing TSC^36^.

Non‐nodular protrusions of varying shape and size along the ventricular wall signal acute or subacute IVH. When the periventricular parenchyma is involved, as in PVHI, subsequent tissue loss may appear over time as wedge‐shaped or round indentations^37^, ^38^. Alternatively, a deep wedge‐shaped indentation can correspond to the ventricular end of a closed‐lip cleft (schizencephaly), reflecting the sequela of an early parenchymal insult^39^.

Non‐nodular protrusions or round indentations accompanied by ependymal abnormalities can be seen in intrauterine CMV infection. In this context, protrusions reflect ependymal adhesions, whereas indentations correspond to periventricular white‐matter loss. The accompanying pattern of abnormalities, such as a periventricular ‘halo’ of echogenicity, subependymal cysts, periventricular calcifications and abnormal lamination, illustrates the extent and distribution of parenchymal involvement^40^, ^41^, ^42^.

Mixed or undulating LVBI patterns can be either focal or diffuse, reflecting variability in both the timing and severity of underlying insults. Diffuse configurations, often identified in midgestation, were frequently observed with MCD and other CNS anomalies, suggesting an early insult that alters neuronal migration and organization. Histologically, these probably correspond to a combination of periventricular and subcortical heterotopia that distorts the ventricular contour^11^, ^31^. In contrast, focal mixed or undulating LVBI patterns are more consistent with later‐onset brain injury such as PVHI‐IVH, representing localized parenchymal injury superimposed on otherwise preserved architecture.

### Limitations

Our study has several limitations. First, there is a potential for selection bias owing to the tertiary referral setting. The cohort may not fully represent the broader population of fetuses with LVBI, especially those with milder or transient findings that might not prompt referral. Although other MRI sequences such as T1, T2* and diffusion‐weighted imaging can provide complementary information, our analysis was limited to T2‐weighted imaging (the most informative sequence for structural assessment of the fetal brain).

A major limitation of the study is the limited and heterogeneous nature of the available genetic and neurological outcome data, with over half of the cohort lacking genetic confirmation. This reflects both the retrospective design of the study and the evolving accessibility of advanced genetic diagnostics during the study period. Neurological outcome data were available for only 42.4% of the cohort, largely owing to the high rate of TOP (50.0%). Although this cohort is the largest reported to date to our knowledge, subdivision into six morphological subgroups resulted in small numbers for subgroup analysis. The reproducibility of our LVBI pattern classification was evaluated only within an expert panel; interobserver agreement among general sonographers was not assessed, and borderline cases may prove challenging to categorize in routine practice. Therefore, these findings should not yet be applied to clinical counseling or decision‐making.

It is important to emphasize that the present study is descriptive. The observed LVBI configurations should not be used in isolation to infer genetic or prognostic implications. Further prospective studies with complete genetic testing and standardized longitudinal follow‐up are needed before any clinical application can be considered.

### Conclusion

When the fetal lateral ventricles exhibit dilation or contour irregularities, systematic characterization is warranted. Recognizing and classifying LVBI according to the proposed morphological patterns may assist in interpreting the possible underlying developmental or disruptive etiology and establish a common descriptive framework for future research and outcome studies.

## Supporting information


**Table S1** Prenatal imaging characteristics, associated findings and outcomes in fetuses with nodular protrusion patterns of lateral ventricular border irregularities.

**Table S2** Prenatal imaging characteristics, associated findings and outcomes in fetuses with non‐nodular protrusion patterns of lateral ventricular border irregularities.

**Table S3** Prenatal imaging characteristics, associated findings and outcomes in fetuses with wedge‐shaped indentation patterns of lateral ventricular border irregularities.

**Table S4** Prenatal imaging characteristics, associated findings and outcomes in fetuses with round indentation patterns of lateral ventricular border irregularities.

**Table S5** Prenatal imaging characteristics, associated findings and outcomes in fetuses with undulation patterns of lateral ventricular border irregularities.

**Table S6** Prenatal imaging characteristics, associated findings and outcomes in fetuses with mixed patterns of lateral ventricular border irregularities.

**Table S7** Mean, median and range of gestational age at time of first neurosonogram for each pattern of lateral ventricular border irregularities.

## Data Availability

The data that supports the findings of this study are available in the supplementary material of this article.
