# Supplementary material for: Morphological patterns of fetal lateral ventricular border irregularities: descriptive study
Source: Ultrasound Obstet Gynecol. 2026 Apr 15;67(5):635–45. doi: 10.1002/uog.70217 (PMC13136058; doi:10.1002/uog.70217)
Supplement: Supplementary file 1 — Table S1 Prenatal imaging characteristics, associated findings and outcomes in fetuses with nodular protrusion patterns of lateral ventricular border irregularities. [file UOG-67-635-s001.docx]

Table S1: Pattern 1- Nodular protrusions (NP)

| Case no | GA/ Gender | US pattern of LVBI | MRI | Lateral ventricles | Additional findings | Prenatal testing | Prenatal diagnosis | Outcome |
| --- | --- | --- | --- | --- | --- | --- | --- | --- |
| NP1 | 36+6  Female | Multiple contiguous small diffuse nodules, intermediate echogenicity | T2 hypointense multiple contiguous nodules | Square dilated anterior horns | Enlarged posterior fossa | CMA- normal | Diffuse PNH, susp. Filamin A mutation | No definitive diagnosis  Normal development at 1 year |
| NP2 | 31+5  Female | Multiple contiguous small diffuse nodules, intermediate echogenicity | T2 hypointense multiple contiguous nodules | Square dilated anterior horns | Enlarged posterior fossa | - | Diffuse PNH, susp. Filamin A mutation | TOP (at another center)  Maternal Filamin A mutation |
| NP3 | 25+0  Female | Multiple small diffuse nodules, intermediate echogenicity & Undulation | T2 hypointense multiple contiguous nodules  (Postnatal MRI) | Square anterior horns | Posterior fossa arachnoid cyst | - | Diffuse PNH, susp. Filamin A mutation | Filamin A mutation (de novo)  Normal development at 6 months |
| NP4 | 26+2  Female | Multiple diffuse & Undulation | T2 hypointense contiguous nodules | Square dilated anterior horns | Enlarged posterior fossa with suspected arachnoid cyst, VSD | Maternal MRI – PNH (Epileptic mother) | M/p Filamin A mutation | Filamin A mutation  Normal development  2.8 year, susp. single epileptic episode |
| NP5 | 29  Female | Multiple diffuse small nodules, with intermediate echogenicity & Undulation, serrated ependyma | T2 hypointense multiple nodules, thick, Undulation, and serrated ependyma | Bilateral severe and progressive ventriculomegaly | Disrupted septum pellucidum | - | Hydrocephalus d/t aqueductal stenosis, PNH  Disruptive / Neurogenetic etiology | VP shunt insertion at 2 weeks  PNH m/p disruptive etiology (IVH)  Normal development at 27 months |
| NP6 | 22+5  Male | Multiple asymmetric nodules, right > left, homogenous in size with intermediate echogenicity & Undulation | T2 hypointense multiple periventricular nodules, mostly temporal | Prominent ventricles, atypical right anterior horn | - | CMA & WES- normal  (AFP = 16 MOM in amniotic fluid) | Maternal single nodules | ASD, ODD, ADHD, language delay at 6 years, normal intelligence  No definitive diagnosis |
| NP7 | 31+0 | Multiple asymmetric, atrial, and posterior nodules with intermediate echogenicity | T2 hypointense multiple periventricular nodules | - | Abnormally oriented deep, irregular posterior sulci | - | Susp. PNH and PMG | TOP |
| NP8 | 31+2  Female | Multiple asymmetric nodules, with intermediate echogenicity | T2 hypointense multiple periventricular nodules (mostly parieto-occipital on one side) | Bilateral mild ventriculomegaly, dysmorphic anterior horns | Premature ipsilateral parietal sulcation, MCM | - | PNH & MCD  Disruptive / Neurogenetic etiology | TOP  PM- multiple bilateral PNH, abnormal gyral pattern (dysgyria), hypoplastic CC, facial dysmorphism |
| NP9 | 22+4 | Multiple asymmetric bilateral parietal nodules with intermediate echogenicity and undulation borders, serrated ependyma | - | Enlarged asymmetric & dysmorphic ventricles | Deviated IH fissure and CSP  Cleft palate | - | PNH  Susp. neurogenetic etiology | TOP |
| NP10 | 26+3  Male | Multiple asymmetric anterior nodules with intermediate echogenicity | - | Bilateral mild ventriculomegaly | CC agenesis, IH cyst, delayed sulcation, abnormal cerebellum and vermis, complex cardiac anomaly, facial dysmorphism, curly toe | CMA & WES- Variant in MED13L | Mowat Wilson syndrome | PND |
| NP11 | 33+6  Female | Multiple asymmetric nodules, with intermediate echogenicity | Subependymal T2 hypointense nodules | Asymmetric ventriculomegaly | Squared dilated left frontal horn, IUGR | CMA & WES - normal | PNH & IUGR, probably placental origin | TOP, PM -Periventricular and subcortical heterotopia, placental pathology |
| NP12 | 33+0 | Multiple nodules, various sizes, iso and intermediate echogenicity | Subependymal T2 hypointense nodules | Mild bilateral ventriculomegaly | US - Multiple cortical and subcortical echogenic lesions of various size and shape  MRI- multiple cortical and subcortical T2 hypointense lesions  Multiple cardiac Rhabdomyomas | - | TSC | TOP |
| NP13 | 26+5  Female | Multiple various size nodules, iso and intermediate echogenicity | T2 hypointense multiple subependymal nodules | - | US - Multiple cortical and subcortical echogenic lesions of various size and shape  MRI- multiple cortical and subcortical T2 hypointense lesions  Two cardiac rhabdomyomas | - | TSC | TOP, PM - Multiple cardiac rhabdomyomas, subependymal nodules, foci of cerebellar cortical dysplasia, possible early tubers |
| NP14 | 31+1  Female | Multiple various size nodules, iso and intermediate echogenicity | - | - | US- Multiple cortical and subcortical echogenic lesions of various size and shape  Multiple cardiac rhabdomyomas | CMA normal | TSC | TOP  PM- Multiple cardiac rhabdomyomas, subependymal nodules, multiple foci of cerebellar cortical dysplasia- tubers |
| NP15 | 25+5  Male | Multiple various size nodules, iso and intermediate echogenicity | T2 hypointense multiple subependymal nodules | Asymmetric ventricles | US- Multiple cortical and subcortical echogenic lesions of various size and shape  MRI- multiple cortical and subcortical T2 hypointense lesions  Two cardiac rhabdomyomas | - | TSC | Live birth |
| NP16 | 31+0  Female | Multiple various-size nodules, iso, and intermediate echogenicity | - | - | Multiple cortical and subcortical echogenic lesions of various size and shape  Four cardiac rhabdomyomas | - | TSC | Epilepsy diagnosed at 2 years, normal development at 7 years  Resection of cardiac lesion obstructing LVOT at 5 years, pathology -rhabdomyoma |
| NP17 | 32+6  Female | Single (**≤** 4) anterior and posterior bilateral nodules, intermediate echogenicity | - | Bilateral ventriculomegaly, square anterior horns | Unilateral clubfoot | - | PNH  Disruptive / Neurogenetic etiology | IUFD (35GA) |
| NP18 | 35+4  Female | Single (**≤** 4) left occipital nodules, intermediate echogenicity | T2 hypointense, single left occipital nodules | Prominent, asymmetric ventricles | - | - | PNH- m/p idiopathic | Normal development at 6 years |
| NP19 | 25+6  Male | Single (< 4) right parietal nodules, intermediate echogenicity | T2 hypointense, single right parietal nodules | Prominent right ventricle, asymmetry | Deviated CSP | - | PNH- m/p idiopathic | LFU |

**Abbreviations:**
ASD, Autism Spectrum Disorder; CC, Corpus Callosum; CMA, **Chromosomal Microarray Analysis; CSP, Cavum Septum Pellucidum; GA, Gestational Age; IH, Interhemispheric ; IUFD,** Intra Uterine Fetal Death; **IUGR,** Intra Uterine Growth Restriction**;** LFU, Lost to Follow-Up; LVBI, Lateral Ventricular Border Irregularity; LVOT, Left Ventricle Outflow Tract; MCD, Malformation of Cortical Development; MCM, Mega Cisterna Magna; MRI, Magnetic Resonance Imaging; ODD, Oppositional Defiant Disorder, PM, Postmortem; PND, Perinatal Death; PNH, Periventricular Nodular Heterotopia; TOP, Termination of Pregnancy; TSC, Tuberous Sclerosis Complex; US, Ultrasound; VP, ventriculoperitoneal; WES, Whole Exome Sequencing.
