# Supplementary material for: Morphological patterns of fetal lateral ventricular border irregularities: descriptive study
Source: Ultrasound Obstet Gynecol. 2026 Apr 15;67(5):635–45. doi: 10.1002/uog.70217 (PMC13136058; doi:10.1002/uog.70217)
Supplement: Supplementary file 2 — Table S2 Prenatal imaging characteristics, associated findings and outcomes in fetuses with non‐nodular protrusion patterns of lateral ventricular border irregularities. [file UOG-67-635-s005.docx]

Table S2: Prenatal imaging characteristics, associated findings and outcomes in fetuses with non-nodular protrusion (NNP) patterns of lateral ventricular border irregularities.

| Case number | GA/ Gender | US pattern of LVBI | MRI | Lateral ventricles | Additional findings | Prenatal testing | Prenatal diagnosis | Outcome |
| --- | --- | --- | --- | --- | --- | --- | --- | --- |
| NNP1 | 36+5  Female | Multiple bilateral non-nodular hyperechogenic protrusions, thick echogenic ependyma | Multiple bilateral non-nodular protrusions, periventricular blood remnants | Severe bilateral ventriculomegaly | - | - | IVH grade III | Post hemorrhagic hydrocephalus, VP shunt insertion with normal development at 24 months |
| NNP2 | 24+0  Male | Multiple non-nodular Hyperechogenic protrusions, left ventricle, hyperechogenic ependyma  Resolution at 28 GA | Multiple non-nodular protrusions, periventricular blood remnants | Asymmetric ventriculomegaly left >> right | - | - | IVH grade II | Normal development at 2 years |
| NNP3 | 35+6  Female | Single non-nodular hyperechogenic protrusion, hyperechogenic ependyma | Single non-nodular protrusion, periventricular blood remnants | Asymmetric ventriculomegaly left >> right | - | Normal CMA | IVH grade II | Normal development at 4 years |
| NNP4 | 23+6  Male | Single non-nodular hyperechogenic protrusion, hyperechogenic ependyma | - | Occipital horn adhesions, periventricular calcifications | Echogenic bowel, splenomegaly | - | CMV (Positive amniotic PCR- CMV) | TOP |

**Abbreviations:**
CMA, **Chromosomal Microarray Analysis; CMV, Cytomegalovirus**; **GA, Gestational Age; IVH, Intraventricular Hemorrhage;** LVBI, Lateral Ventricular Border Irregularity; MRI, Magnetic Resonance Imaging; PCR, Polymerase Chain Reaction; TOP, Termination of Pregnancy; US, Ultrasound; VP, ventriculoperitoneal
