# Supplementary material for: Morphological patterns of fetal lateral ventricular border irregularities: descriptive study
Source: Ultrasound Obstet Gynecol. 2026 Apr 15;67(5):635–45. doi: 10.1002/uog.70217 (PMC13136058; doi:10.1002/uog.70217)
Supplement: Supplementary file 3 — Table S3 Prenatal imaging characteristics, associated findings and outcomes in fetuses with wedge‐shaped indentation patterns of lateral ventricular border irregularities. [file UOG-67-635-s003.docx]

Table S3: Pattern 3 - Wedged indentations (WI)

| Outcome | Prenatal diagnosis | Prenatal testing | Additional findings | Lateral ventricles | MRI | US pattern of LVBI | GA/ Gender | Case number |
| --- | --- | --- | --- | --- | --- | --- | --- | --- |
| Normal development at 4.5 years | IVH- PVHI | Karyotype- normal | - | Dilated right anterior horn | Right posterior frontal wedged indentation (small shallow single) | Right posterior frontal wedged indentation (small shallow single) with hyperechogenic borders | 31+5  Female | WI1 |
| Normal development at 5 years | IVH- PVHI | - | Bilateral CPC with T2 hypointense borders  Outer cerebellar irregular contour with T2 hypointense borders | Asymmetric mild ventriculomegaly, dilated anterior horns | Left parietal wedged indentation (small shallow), T2 hypointense border | Left parietal wedged indentation (small shallow single) with hyperechogenic borders | 32+0  Male | WI2 |
| Normal development at 7 years | IVH- PVHI | CMA- normal | - | Asymmetric ventriculomegaly right | - | Right parietal wedged indentation (small single) with hyperechogenic borders | 34+0  Male | WI3 |
| Normal development at 3 years | IVH- PVHI | CMA- normal | - | Asymmetric ventriculomegaly  right | - | Right frontal wedged indentation (small single) with hyperechogenic borders | 24+6  Male | WI4 |
| TOP,  PM- left parietal PVHI | IVH- PVHI | - | Multiple porencephalic cysts in the adjacent periventricular parenchyma  MRI- basal ganglia involvement | Asymmetric ventriculomegaly left | Wedged indentation, (small single), periventricular blood remnants | Left parietal  wedged indentation (small single) with hyperechogenic borders | 38+3  Male | WI5 |
| TOP,  PM- diffuse white matter injury, periventricular cystic spaces, hemosiderin macrophages and minute calcifications. LGA fetus, placental insufficiency. | IVH- PVHI | CMA & WES- normal | Multiple porencephalic cysts left posterior frontal lobe,  blurring of cerebellar outer margins. | Asymmetric severe ventriculomegaly left | Hyperintense signal bordering porencephalic cysts | Left posterior frontal wedged indentation, single | 34+5  Male | WI6 |
| LFU | PVHI | CMA- normal | Asymmetric CC, deviated CSP | Mild ventriculomegaly asymmetric right,  dysmorphic right anterior horn | No abnormal findings | Right frontal  wedged indentation, single with hyperechogenic borders | 32+3  Male | WI7 |
| Normal development at 4 years | IVH- PVHI | CMA- normal | - | Mild ventriculomegaly asymmetric left | Left periventricular blood remnants, Tractography- Normal CST | Left fronto-parietal wedged indentation, single, with hyperechogenic borders | 35+6  Female | WI8 |
| TOP | IVH - PVHI | CMA  WES – COL4A1 de novo mutation | Decreased size right crus cerebri and thalamus | Mild ventriculomegaly asymmetric right | Right parietal indentation, periventricular blood remnants  Tractography- right CST involvement | Right parietal wedged indentation, with hyperechogenic serrated ependyma | 33+2  Male | WI9 |
| Profound intellectual disability, intractable epilepsy, CVI, cerebral palsy | PVHI  Rubinstein - Taybi syndrome | CMA & WES – CREBBP de novo mutation | Blood remnants in the left ventricle lumen | Asymmetric ventriculomegaly (left)  Dysmorphic anterior horn (left) | - | Left frontal wedged indentation and hyperechogenic serrated ependyma | 32+4  Male | WI10 |
| TOP,  PM-  Left cerebral hemisphere - white matter & subcortical nodular heterotopia, focal irregular gyration.  Right cerebral hemisphere - focal abnormal cortical thickening. Loss of normal layering | MCD,  Susp. hypoxic-ischemic etiology | - | Irregular cortex above LVBI (suspected PMG) | Asymmetric ventriculomegaly lt. | - | left frontal wedged indentation | 25+6 | WI11 |
| Microcephaly,  dysmorphic features,  m/p genetic vascular etiology  Severe GDD, epilepsy, hypotonia at 2 years | Bilateral PVHI    Susp. genetic- vascular etiology | Normal CMA | Left hemisphere diffusion restriction on MRI,  multiple  vascular malformations,  polyhydramnios | Asymmetric ventriculomegaly,  unilateral squared shape and irregular anterior horn | Bilateral fronto-parietal wedged indentations periventricular blood remnants | Bilateral multiple fronto-parietal wedged indentations | 33+3  Female | WI12 |
| Septo-optic dysplasia plus (associated with schizencephaly and PMG)  Normal development at 6 years, growth hormone deficiency, susp. epilepsy | Closed lip schizencephaly with susp. right peri-Sylvian PMG | - | Closed lip schizencephaly overlying LVBI, SP agenesis, irregular right peri-Sylvian and frontal cortex (susp. PMG)  Thin optic nerves | Point down appearance of the anterior horns on the coronal view | Right posterior frontal  wedged indentation | Right posterior frontal  wedged indentation with serrated ependyma | 32+4  Female | WI13 |
| Selective termination | Schizencephaly and diffuse white matter damage due to early ischemic event, a complication of MC twins | CMA - normal | Closed lip schizencephaly overlying LVBI,  periventricular white matter hyperechogenicity and calcifications, bilateral, parieto-temporal | Squared shape anterior horns | bilateral frontal wedged indentations | left frontal wedged indentation, hyperechogenic borders | 26+2  Female (MC BA twins) | WI14 |
| Live birth | Schizencephaly | CMA & WES- normal | Closed lip schizencephaly overlying LVBI | - | Right parietal wedged indentation | Right parietal wedged indentation, hyperechogenic borders | 33+6  Male | WI15 |

Abbreviations:
CC, Corpus Callosum; CMA, **Chromosomal Microarray Analysis; CPC, Choroid Plexus Cyst; CSP, Cavum Septum Pellucidum; CST, corticospinal tract; CVI, Cortical Visual Impairment;** GDD, Global Developmental Delay; **GA, Gestational Age; IVH, Intraventricular Hemorrhage**; LGA, Large For Gestational Age; LFU, Lost to Follow-Up; LVBI, Lateral Ventricular Border Irregularity; MC BA, Monochorionic Biamniotic; MCD, Malformation of Cortical Development; MRI, Magnetic Resonance Imaging; PM, Postmortem; PMG, Polymicrogyria; PVHI, Periventricular Hemorrhagic Infarction; SP, Septum Pellucidum; TOP, Termination of Pregnancy; US, Ultrasound; WES, Whole Exome Sequencing.
