# Supplementary material for: Morphological patterns of fetal lateral ventricular border irregularities: descriptive study
Source: Ultrasound Obstet Gynecol. 2026 Apr 15;67(5):635–45. doi: 10.1002/uog.70217 (PMC13136058; doi:10.1002/uog.70217)
Supplement: Supplementary file 4 — Table S4 Prenatal imaging characteristics, associated findings and outcomes in fetuses with round indentation patterns of lateral ventricular border irregularities. [file UOG-67-635-s007.docx]

Table S4: Pattern 4 - Round indentations (RI)

| Outcome | Prenatal diagnosis | Prenatal testing | Additional findings | Lateral ventricles | MRI | US pattern of LVBI | GA/ Gender | Case number |
| --- | --- | --- | --- | --- | --- | --- | --- | --- |
| Normal development at 2.5 years | Porencephalic PVHI | - | Left SP disruption, blurred cerebellar contour,  muscular VSD | Asymmetric ventriculomegaly | Periventricular parenchymal indentations | Left anterior frontal round, hyperechogenic indentations and  Serrated ependyma | 29+2  Female | RI1 |
| Left-hand preference at 4.5 months.  Normal development at 1.5 years | Porencephalic PVHI | CMA & WES -normal | SP deviation | Asymmetric ventriculomegaly | Left frontal porencephalic periventricular cysts communicating with the ventricular lumen | Left frontal round, hyperechogenic indentations and  serrated, thick ependyma | 38+4  Male | RI2 |
| TOP, PM-  white matter necrosis, hydrocephalus, PVHI | IVH-PVHI (Porencephalic) & hydrocephalus | CMA- normal | Hydrocephalus | Bilateral severe ventriculomegaly, dysmorphic anterior horns | Right frontal porencephalic periventricular cyst | Right frontal round hyperechogenic indentations and  serrated ependyma | 38+1  Male | RI3 |
| LFU | Extensive Porencephalic PVHI with basal ganglia involvement | - | Ipsilateral basal ganglia involvement | Asymmetric ventriculomegaly, dysmorphic left anterior horn | - | Extensive left frontal round hyperechogenic indentations,  thick ependyma | 23+0  Female | RI4 |
| TOP | Periventricular cysts  CMV-positive in PCR from amniotic fluid | - | Abnormal lamination | Asymmetric ventriculomegaly | - | Parietooccipital round (2)  hyperechogenic indentations,  thick ependyma | 22+4  Female | RI5 |
| TOP    CMV  Positive PCR in fetal blood | Periventricular cysts  CMV or pseudo-TORCH | - | Abnormal sulcation,  deviated CSP, hepatomegaly | Asymmetric ventricles, dysmorphic dilated left anterior horn, periventricular calcifications | Multiple periventricular cysts | Round indentations,  diffuse,  serrated ependyma | 34+1  Male | RI6 |
| TOP  CMV | Periventricular cysts  CMV or pseudo-TORCH | - | Parenchymal loss, cerebellar hypoplasia | Asymmetric dysmorphic ventricles, periventricular calcifications | - | Round, hyperechogenic indentations,  thick, serrated ependyma | 22+4  Female | RI7 |

**Abbreviations:**
CMA, **Chromosomal Microarray Analysis; CMV, Cytomegalovirus; CNS, Central Nervous System; CSP, Cavum Septum Pellucidum; GA, Gestational Age; IVH, Intraventricular Hemorrhage;** LFU, Lost to Follow-Up; LVBI, Lateral Ventricular Border Irregularity; MRI, Magnetic Resonance Imaging; PCR, Polymerase Chain Reaction; PM, Postmortem; PVHI, Periventricular Hemorrhagic Infarction; SP, Septum Pellucidum; TOP, Termination of Pregnancy; US, Ultrasound; VSD, Ventricular Septal Defect; WES, Whole Exome Sequencing.
