# Supplementary material for: Morphological patterns of fetal lateral ventricular border irregularities: descriptive study
Source: Ultrasound Obstet Gynecol. 2026 Apr 15;67(5):635–45. doi: 10.1002/uog.70217 (PMC13136058; doi:10.1002/uog.70217)
Supplement: Supplementary file 5 — Table S5 Prenatal imaging characteristics, associated findings and outcomes in fetuses with undulation patterns of lateral ventricular border irregularities. [file UOG-67-635-s004.docx]

Table S5: Prenatal imaging characteristics, associated findings and outcomes in fetuses with undulation (U) patterns of lateral ventricular border irregularities.

| Outcome | Prenatal diagnosis | Prenatal testing | Additional findings | Lateral ventricles | MRI | US pattern of LVBI | GA/ Gender | Case number |
| --- | --- | --- | --- | --- | --- | --- | --- | --- |
| TOP | Complex MCD,  Disruptive / neurogenetic etiology  (tubulinopathy?) | CMA- normal | Susp. bilateral asymmetric PMG, basal ganglia asymmetry, distorted IHF, partial agenesis of CC, dysgenetic cerebellum and brain stem | Asymmetric ventriculomegaly, dilated anterior horns | Diffuse bilateral undulation | Diffuse bilateral undulation with hyperechogenic serrated ependyma | 28+6  Male | U1 |
| TOP  PM – disorganized cortex, cobblestone malformation, foci of heterotopia | Susp. cobblestone malformation | - | Asymmetric  thin hemispheres, abnormal lamination,  irregular cortex, Z- shape brain stem,  bilateral club foot,  IUGR | Asymmetric ventriculomegaly | - | Diffuse bilateral undulation | 18+3  Male | U2 |
| FLVCR2 mutation causing Fowler proliferative vasculopathy (postnatal WES)  Severe developmental delay at 2.5 years, intractable epilepsy | Susp. Interferonopathy | - | Periventricular and thalamic calcifications,  microcephaly, parenchymal atrophy, abnormal sulcation (susp. PMG) | Prominent ventricles,  dilated anterior horns | - | Diffuse bilateral undulation, serrated ependyma | 26+4  Male | U3 |
| TOP | Disruptive / neurogenetic etiology | - | microcephaly, CC agenesis, simplified gyral pattern and suspected PMG,  parenchymal thinning, cerebellar hypoplasia | Colpocephaly,  dysmorphic anterior horns | - | Diffuse undulation | 24+1 | U4 |
| Severe GDD | Disruptive / neurogenetic etiology | - | Partial SP agenesis,  abnormal sulcation,  IUGR, scoliosis, cervical hyperextension, bilateral clubfoot | Prominent dysmorphic lateral ventricles | - | Diffuse undulation | 27+3  Female | U5 |
| TOP,  PM- asymmetry of brain lobes, diffuse polymicrogyria and white matter heterotopia, dysgenetic vermis and cerebellum, polydactyly, facial dysmorphism, micropenis | Susp. Ciliopathy | - | Abnormal lamination and sulcation, asymmetric hemisphere, pontocerebellar hypoplasia,  micropenis | Asymmetric ventriculomegaly dysmorphic | - | Diffuse undulation | 27+6  Male | U6 |
| Severe developmental delay at 1.5 year  Normal postnatal WES | Susp. MCD | - | Diffuse fronto-parietal PMG, small anterior lobes, short long bones | Asymmetric prominent ventricles, dysmorphic anterior horns | - | Fronto-parietal  undulation  right > left | 27+2  Male | U7 |
| TOP | Disruptive / syndromic etiology | Karyotype- normal | Multiple subependymal right cysts  Head growth deceleration, liver portal cyst | Asymmetric prominent ventricles | Undulation | Bilateral parieto-occipital undulation, thick, serrated ependyma | 38+3  Female | U8 |
| TOP  PM – asymmetric ventricles, frontal dysgyria  hypoplastic vermis | Tubulinopathy | CMA & WES-  TUBB3 mutation (inherited from a healthy mother)  Maternal and healthy sibling MRI – similar changes | Asymmetric cerebral hemispheres, IHF interdigitation, Abnormal asymmetric sulcation,  dysplastic vermis, subependymal cysts | Asymmetric (left) ventriculomegaly,  dysmorphic left anterior horn | Left ventricle undulation | Left fronto-parietal undulation | 34+0  Male | U9 |
| Congenital CMV (positive PCR in Guthrie card)  Congenital cataract, vestibular disorder, ataxia at 5.5 years | Unknown etiology | CMA & WES – normal  PCR – CMV negative | subtle periventricular calcifications | Asymmetric prominent ventricles | Minimal irregularity of LVB, no blood products | Left  undulation,  hyperechogenic serrated ependyma | 32+5  Female | U10 |
| Normal development at 3 years | Susp. new neurogenetic syndrome | Maternal MRI- single nodules Sibling MRI- PNH  Very high AFP in amniotic fluid  CMA & WES- normal | - | - | Undulation borders lt > rt | Left undulation | 22+3  Female | U11 |

**Abbreviations:**
CC, Corpus Callosum; CMA, **Chromosomal Microarray Analysis; CMV, Cytomegalovirus; CNS, Central Nervous System; GA, Gestational Age;** GDD, Global Developmental Delay**; IHF, Interhemispheric Fissure; IUGR,** Intra Uterine Growth Restriction**; IVH, Intraventricular Hemorrhage;** LFU, Lost to Follow-Up; LVBI, Lateral Ventricular Border Irregularity; MCD, Malformation of Cortical Development; MRI, Magnetic Resonance Imaging; PCR, Polymerase Chain Reaction; PM, Postmortem; PMG, Polymicrogyria; PNH, Periventricular Nodular Heterotopia; SP, Septum Pellucidum; TOP, Termination Of Pregnancy; US, Ultrasound; VSD, Ventricular Septal Defect; WES, Whole Exome Sequencing.
