# Supplementary material for: Morphological patterns of fetal lateral ventricular border irregularities: descriptive study
Source: Ultrasound Obstet Gynecol. 2026 Apr 15;67(5):635–45. doi: 10.1002/uog.70217 (PMC13136058; doi:10.1002/uog.70217)
Supplement: Supplementary file 6 — Table S6 Prenatal imaging characteristics, associated findings and outcomes in fetuses with mixed patterns of lateral ventricular border irregularities. [file UOG-67-635-s002.docx]

Table S6: Prenatal imaging characteristics, associated findings and outcomes in fetuses with mixed (M) patterns of lateral ventricular border irregularities.

| Outcome | Prenatal diagnosis | Prenatal testing | Additional findings | Lateral ventricles | MRI | US pattern of LVBI | GA/ Gender | Case number |
| --- | --- | --- | --- | --- | --- | --- | --- | --- |
| TOP | Aicardi syndrome | - | Asymmetric hemispheres, ACC, IH cysts, thick elongated tectum, retinal coloboma, severe hydronephrosis | Asymmetric dysmorphic | - | Multiple nodular bulges & round depressions | 24+6  Female | M1 |
| TOP  PM-  occipital skull  defect, PVNH &  subcortical heterotopia, facial dysmorphism  susp. developmental /genetic syndrome | Complex brain malformation | CMA & WES -normal | Atretic posterior encephalocele, abnormal sulcation, thick tectum | Asymmetric dysmorphic | - | Multiple nodular bulges & wedge depressions | 24+4  Female | M2 |
| TOP | Aicardi syndrome | - | ACC, asymmetric cerebellar hemispheres, susp. diffuse PMG, IHF interdigitation and cyst, dysplastic cerebellum and thick tectum, retinal coloboma | Enlarged dysmorphic ventricles | Nodular bulges & wedge depressions | Multiple nodular bulges & wedge depressions, hyperechogenic ependyma | 34+2  Female | M3 |
| TOP | Susp. neurogenetic syndrome | - | Partial ACC, abnormal operculization with susp. Sylvian fissure PMG, cerebellar hypoplasia, vermian agenesis, Tetralogy of Fallot | Asymmetric ventriculomegaly | - | Multiple round depressions with foci of undulation irregularities | 28+1  Female | M4 |
| TOP  PM – thick CC, heterotopia  diffuse PMG  white matter calcifications,  CMV inclusions bodies | Fetal CMV infection | Maternal CMV seroconversion | Abnormal lamination, delayed sulcation, occipital subependymal cysts, cerebellar hypoplasia  Hepato-splenomegaly, early IUGR | Asymmetric ventriculomegaly | - | Multiple round depressions with foci of undulation irregularity, periventricular calcifications, echogenic, thick serrated ependyma | 25+4 | M5 |
| TOP | Fetal CMV infection | Maternal CMV seroconversion | abnormal lamination, parenchymal thinning, abnormal sylvian sulcation, small subependymal cysts, dysgenetic CC, dysgenetic cerebellum, microcephaly, Echogenic bowel | Prominent ventricles | Multiple round depressions with foci of undulation irregularities, abnormal WM signal | Multiple round depressions with foci of undulation irregularities, periventricular calcifications, echogenic, thick serrated ependyma | 24+1  Female | M6 |
| TOP  PM - IUGR asymmetric, diffuse bilateral white matter injury with right frontoparietal PVHI | PVHI | - | Right frontal parenchymal hyper echogenicity with small porencephalic cysts, oligohydramnios | Prominent ventricles | Non-nodular bulges with foci of undulation irregularities  Right fronto-parietal large hemorrhagic infarct | Right frontal non-nodular hyperechogenic bulges with foci of undulation irregularities, hyperechogenic ependyma | 30+0  Male | M7 |
| TOP, PM- IVH PVHI | IVH PVHI | CMA -normal | Hyperechogenicity of the right fronto-parietal periventricular parenchyma  Bilateral non- homogenous choroid plexus and blood clots in the ventricular lumen | Ventriculomegaly | - | Right fronto-parietal non nodular hyperechogenic bulges with foci of undulation irregularities, hyperechogenic, serrated ependyma | 28+3 | M8 |
| CP quadriplegia asymmetric, GMFCS 3,  Borderline intelligence, CVI at 5 years | PVHI | CMA -normal  FNAIT (Maternal anti-HPA15 antibodies) | Parenchymal porencephalic cyst, hydrops, severe fetal anemia | Mild bilateral ventriculomegaly | Multiple non-nodular bulges, periventricular blood remnants | Parietal and internal capsule wedge depressions, multiple non-nodular hyperechogenic bulges with serrated, thick ependyma | 33+1  Male | M9 |
| TOP  PM – dysmorphic right lateral ventricle,  areas of thick cortex- susp. cortical dysplasia. No blood remnants | Susp. disruptive event | - | - | Prominent ventricles, square dilated anterior right horn | Right frontal nodular irregularities with T2 low signal | Right frontal round depressions with foci of undulation irregularities. hyperechogenic, thick, serrated ependyma | 26+5  Male | M10 |

**Abbreviations:**

ACC, Agenesis of Corpus Callosum; CC, Corpus Callosum; CMA, **Chromosomal Microarray Analysis; CMV, Cytomegalovirus; CNS, Central Nervous System; CP, Cerebral palsy; CVI, Cortical Visual Impairment; FNAIT, Fetal and Neonatal Alloimmune Thrombocytopenia; GA, Gestational Age; IH, Interhemispheric; IHF, Interhemispheric Fissure; IUGR,** Intra Uterine Growth Restriction; **IVH, Intraventricular Hemorrhage;** LVBI, Lateral Ventricular Border Irregularity; MRI, Magnetic Resonance Imaging; PM, Postmortem; PMG, Polymicrogyria; PVHI, Periventricular Hemorrhagic Infarction; PVNH, Periventricular Nodular Heterotopia; TOP, Termination of Pregnancy; US, Ultrasound; WES, Whole Exome Sequencing; WM, White Matter

(
