# Supplementary material for: Morphological patterns of fetal lateral ventricular border irregularities: descriptive study
Source: Ultrasound Obstet Gynecol. 2026 Apr 15;67(5):635–45. doi: 10.1002/uog.70217 (PMC13136058; doi:10.1002/uog.70217)
Supplement: Supplementary file 7 — Table S7 Mean, median and range of gestational age at time of first neurosonogram for each pattern of lateral ventricular border irregularities. [file UOG-67-635-s006.docx]

| **LVBI pattern** | **Number of cases** | **Mean GA** | **Median GA** | **Range** |
| --- | --- | --- | --- | --- |
| **Nodular protrusions** | 19 | 29+3 | 31+0 | 22+5 – 36+6 |
| **Non-nodular protrusions** | 4 | 30+1 | 29+6 | 23+6 – 36+5 |
| **Wedged indentations** | 15 | 32+1 | 32+4 | 24+6 – 38+3 |
| **Round indentations** | 7 | 29+5 | 29+2 | 22+4 – 38+4 |
| **Undulation** | 11 | 28+0 | 27+3 | 18+3 – 38+3 |
| **Mixed** | 10 | 28+0 | 27+3 | 24+1–34+2 |
| **Overall cohort** | 66 | 29+5 | 29+4 | 18+3 – 38+4 |

Table S7: Mean, median, and range of gestational ages (in weeks + days) at the time of first neurosonography for each LVBI. GA, gestational age; LVBI, Lateral Ventricular Border Irregularity
